# Clinical Studies with Large Nou-Emetic Doses of Ipecacuanha: With a Contribution to the Therapeusis of Cholera

**Published:** 1875-03

**Authors:** Alfred A. Woodhull

**Affiliations:** Assistant Surgeon U. S. Army


ATLANTA

MEDICAL AND SURGICAL JOURNAL.

VOL. XII.] MARCH—1875. [No. 12.

### Original Communications.

### CLINICAL STUDIES WITH LARGE NON-EMETIC DOSES OF IPECACUANHA: With a Contribution to the Therapeusis of Cholera.

(Continued from page 673 of February number.)

BY ALFRED A. WOODHULL, M.D., ASSISTANT SURGEON U. S. ARMY.

*A Special Report to the Surgeon General U. S. Army, read before the Atlanta Academy of Medicine.*

The following case certainly shows that twenty grains of ipecacuanha may be readily retained in some instances, and probably illustrates its remedial effects in occasional forms of abdominal pain.

CASE XIX.—M. F., of good general health and habits, admitted hospital 27th August, 1874. It appears that late on the night of the 25th he indulged in wine, confectionery and other unaccustomed food, and on the 26th had, between 4 and 5:30 A.M., three sharply painful, thin operations from the bowels, coming on with much suddenness and severity. 6 A.M., 26th—Given magnes. sulph. ℥ iv., magnes., sod. bicarb., aa. ℈ j, pulv. rhei., pulv. zing., aa. gr. v., aq. menth.p., q. s. His bowels moved freely between 8 and 10 A.M., and again several times about 4 P.M., and he also made several ineffectual efforts.

27th.—Bowels moved once in the night, and again, with pain, griping and quickness of action at 5 A.M. Taken into hospital. 11 A.M.—Bowels have not moved since five o'clock, but he has a feeling of great fulness, heaviness and painful oppression in the lower abdomen. Tongue a little coated. Put to bed, and given pulv. ipecac. gr. xx., opii gr. j., in pills. No mustard was used.

A little nausea but no vomiting followed, the abdominal uneasiness quickly disappeared, and he felt quite well in an hour or two. Arose at five o'clock and took supper.

28th.—Remained free from all pain and inconvenience, and was returned to duty.

The three cases immediately succeeding illustrate the ease with which the ipecacuanha frequently is retained, and the slight nausea that happens even when there is emesis; and I think that the change in the character of the stools and in the accompanying sensations may fairly be attributed to it.

CASE XX.—C. S. This man had been ill several weeks with a very mild and open attack of enteric fever. For some days he had had marked looseness of the bowels, generally having two alvine discharges in the night, and three or four in the day. These gave no special pain.

29th August, 1874.—He reports that for the last thirty-six hours he has had very severe and increasing pain with each passage, that the stools had become more consistent, and that he has had sharp suffering extending across the abdomen near the umbilicus, while the actual motion has been accompanied by excruciating pain (the description reminding one of ulcer of the rectum,) and has been followed by tenesmic straining. He has also had several ineffectual promptings to empty the bowels, with straining. 6 P.M.—Gave ipecac. gr. xx., opii gr. j., in pills.

30th.—Felt a little nausea soon after taking the medicine, and again about 8:30 P.M., upon getting up to urinate, but neither sensation was severe, and no sinapism was required. Slept perfectly well from taps to reveille, which he had not before done during his illness. Had a copious, foamy, light yellow dejection, without pain and with but very little discomfort, at 11 A.M. Had no other stool during the day. 9 P.M.—Gave ipecac. gr. x., opii gr. j., in pills.

31st.—Again slept well all night and had no operation through the night or to-day. No abdominal pain.

1st September.—Had one copious, painless passage, similar to the one of 30th, in the night, and another at 7 A.M. Slept well and feels materially better in all respects.

None of the symptoms for which the ipecacuanha was given in this case returned.

CASE XXI.—B. T. A., in the last and convalescent stage of a mild but well-marked attack of typhoid fever. Complains that for several days his stools have been costive, and that he has had great distress and pain when the solid, scanty dejections were voided with straining.

31st August, 1874.—Gave experimentally, at 3 P.M., ipecac. gr. xx., opii gr. j., in pills. 7 P.M.—Has not been nauseated, except when lying on the left side; feels very comfortable.

1st September.—At nine o'clock last night had a severe, cramp-like pain in the stomach, which was relieved by a sinapism, but there was no nausea. Bowels not moved, either in the night or day. 9 P.M.—Gave tr. opii *m*.xv., and fifteen minutes later ipecac. gr. xx., in pills.

2d.—Felt no nausea last night, and went to sleep promptly. Was awakened at 10:30 P.M. by vomiting, without preceding nausea. "Threw up less than a pint of sour or bitter fluid. It wasn't much. Don't know what made me vomit. Didn't feel sick, but I waked up vomiting. Didn't feel sick afterwards. Went to sleep pretty soon." He slept the remainder of the night. As his bowels had not yet moved, gave him ol. ricini f.℥j. at 10 A.M. (It is possible that the vomiting was due to the mode of administration. The tincture may have expended its local influence before the pills dissolved.) His bowels moved three times in the course of the day after the oil was given, the first passage being of natural consistence, and the other two thin. None gave pain. There was no further intestinal pain in this case.

CASE XXII.—P. N., has been feverish and has had severe abdominal pain, with flatulence, but with neither diarrhœa nor constipation, for several days.

1st September, 1874.—Nearly all this morning had severe pains, starting from the region of the bladder and passing toward the umbilicus. About noon they lessened a little, and at 2 P.M. he took ipecac. gr. xx., opii gr. j., in pills. He had no nausea, but the pain in the bowels required a sinapism near the umbilicus. Temperature at half past five, 100° F. About this time the pains lessened materially, and he fell asleep and perspired gently; took opium and quinine in combination at tattoo, and slept fairly all night.

2d.—The pain returned about 7 A.M. Drank a little tea, and then vomited moderately. Had pain all the morning, again diminishing toward noon. At 12:30 P.M. took ipecac. gr. xx., opii gr. j., in pills. Had no nausea in any degree. 6 P.M.—Temperature 102° F.; bowels moved naturally morning and evening.

9 P.M.—Took ipecac., gr. xxv., opii gr. j., in pills, and, during the night, pills of quinine and opium.

3d.—Had no nausea whatever and no pain in the bowels last night. Had one painless, natural operation in the morning.

It would appear that the ipecacuanha here exerted some positive aperient effect, for between noon of one day and sunrise of the next this man took in combination three grains of opium, which manifested itself in a feeling of fulness in the head, and by other disagreeable sensations, and yet he had a natural dejection in the evening and morning. His abdominal troubles ceased and his other symptoms were treated by other means. *See Case XXIV.*

CASE XXIII.—D. K., aged seven years, an officer's son. When in health a robust and vigorous boy, was, after a week's sharp attack of remittent fever, left with a diarrhœa that assumed a dysenteric tendency. There were from six to eight stools daily, and after each there was a small mucoid discharge, generally mingled with blood and always passed with straining. There were also occasional tenesmic feelings and ineffectual efforts at stool. I am told that on two previous occasions he has been seriously ill with a complaint that commenced in exactly this manner, and obstinately lasted for months, and which, after resisting changes of climate and a great variety of treatment, finally yielded to injections of the acetate of lead.

2d September, 1874—He has been sick in this manner, without treatment, four days. The first passage in the morning is natural, but each successive one through the day becomes worse. He has had six stools to-day, all with straining, with consecutive mucous passages and with some blood, and has also made two ineffectual efforts. In other respects his health, except as he is yet weak from the fever, is fair. 9 P.M.—Took tr. opii c. m. xx., aq. f.℥j., followed in fifteen minutes by pulv. ipecac. gr. x., aq. f.℥j., with a few drops of paregoric to disguise the taste. Upon taking this he at first said "It makes me feel sick," but he almost immediately went to sleep without vomiting or further nausea. He slept undisturbed all night.

3d.—His bowels were moved earlier than usual this morning, and, in all, he had four stools during the day. None of these were painful, there was no straining and no blood was passed, and there were no ineffectual efforts. One passage was accompanied by some mucous slime. 9 P.M.—The medicine was repeated exactly as last night.

4th.—He fell asleep immediately after taking the medicine last night, and slept quietly for an hour and a half, when he awoke vomiting profusely. This was so sudden that there was no time even to procure a vessel, and the discharge was copious, including his tea and perhaps the remains of his dinner. There was no pain, the vomiting was accomplished in one act, there was no subsequent nausea and he slept well the rest of the night. The bowels remained a little loose for a day or two, but not painfully nor unnaturally so; and attention to diet was the only further care.

His parents expressed themselves gratified and greatly surprised at the prompt and favorable termination of this attack, for their previous experience had led them to dread a long-continued and serious illness.

In the following case the good results may be ascribed by some entirely to the emesis. Unquestionably this was of advantage, but it is probable that the intestinal canal was in such a morbid state that the mere removal of the irritating contents of the stomach would not have stopped the discharges. Under any circumstances the case at least shows that a large dose of ipecacuanha may be retained soon after the rejection of another.

CASE XXIV.—P. N., (*see Case XXII.*) has been indisposed for some days, although without any special abdominal trouble since 3d inst.

6th September, 1874.—Diarrhœa began about 7 A.M., and about twelve stools were passed before dusk. These were thin, greenish with white specks, and were accompanied by blood in quantities averaging half an ounce. The motions were sometimes painful and sometimes painless. There was, however, constant pain in the lower bowels; he had headache; the tongue was slightly coated; and the temperature at 5:30 P.M. was 99 1-5° F. He had taken through the day small doses of acetate of lead and opium ineffectually. Had eaten nothing except a little milk and some tea. 6:15 P.M.—Took, immediately after a motion, tr. opii ℳ. xxv., and in fifteen minutes pulv. ipecac. ℈ss. in water f ℨij., and used a sinapism. 8 P.M.—Vomited suddenly and without antecedent nausea. The ejected matter was less than a pint, but embraced the half-digested milk and beef that he had eaten the previous day. It was sour and acrid to the taste. 9:30 P.M.—Took ipecac. gr. xx., opii gr. j., in pills. No sinapism.

7th.—The last medicine was retained quietly, and the patient slept well. There was no abdominal pain last night or this morning. There were only two operations in the course of the day, both a little thin but free from pain and with no abnormal appearance.

This man remained practically well during the remainder of September.

The foregoing are all the cases in which large doses of ipecacuanha have been employed at this post during the past summer, except when administered for the distinct and primary purpose of procuring emesis. They are reproduced in detail, (except the long case XIII., which is faithfully epitomized,) in the hope that they will lead to a wider trial of this method, and, especially, that investigation into the confessedly obscure action of this drug may be excited.

As was premised, the successful treatment of dysentery in this manner is common in India; and it may be that it is pursued by a number of physicians in this country. In fact, after this series had well advanced my attention was called to nine cures of that disease by this method, in the summer of 1873, by Dr. Thomas M. Woodson, of Gallatin, Tennessee, (*Amer. Practitioner*, ix., Jan., 1874, p. 31); and after it was completed to another series by Dr. John Stephen, of Pennsylvania (*vide infra*). But that it is not general here is unquestionable.† This is not surprising when we observe the persistent manner in which it has been ignored by systematic teachers of medicine. Maclean is the only writer of this class who has at all developed it. By him its value is clearly and satisfactorily set forth. (Art. Dysentery, Reynolds' *System*; foot-note, Dysentery, Aitken's *Practice*.)

Before its revival in 1858 by Mr. Docker,‡ (7th Fusiliers,) of the British Army, writers of text-books quite naturally would not delve into medical antiquities to treat of obsolete practices or of presumed delusions. But although its efficacy has been so well proven in India, the most of the later writers appear to have thought it inapplicable to other climates. Aitken in his latest edition (*Sci. and Prac.*, 3d Am. fr. 6th Eng. ed., 1872, i., p. 658,) yields

† After this page was in type a note from Dr. C. E. Munn, U. S. Army, informs me that Dr. Bancroft, of the Pennsylvania Volunteers, now of Denver, Colorado, and Dr. DeWolf, 1st Mass. Cav., now of Northampton, Mass., employed this method successfully in treating dysentery as it occurred in their commands in South Carolina [probably in 1863.]

‡ Polichronie, (p. 10) says by M. Segond, of Cayenne. (*See later.*)

to the weight of testimony, and gives in his adhesion to this treatment. But in his earlier editions he merely introduces in a foot-note a letter from Professor Maclean, giving it in the text a half-hearted sentence concluding "but the disease though mitigated is seldom cured by these means."

Niemeyer (Am. fr. 8th Germ. ed., 1871, ii., p. 673,) in an elaborate article upon epidemic dysentery, only refers to ipecacuanha as a preliminary emetic when the stomach is overloaded; and treating of catarrhal (or sporadic) dysentery (i., p. 547,) does not mention it. Trousseau (*Clinical Medicine*, Eng. fr. 3d Fr. ed., 1873, ii., p. 486,) at the beginning of the attack prescribes ipecacuanha in emetic doses, and advises about twelve grains every ten minutes until vomiting is induced, following it that evening or the next day by salines. He seeks emesis for its evacuant effect. Watson (Am. fr. 5th Eng. ed., 1872, ii., p. 581,) briefly outlines the non-emetic method of using large doses, prefacing it with the remark that it "counts many experienced advocates." Dr. Hartshorne, the American editor, adds in a note "more confidence is felt by American practitioners generally in the use of ipecacuanha in small non-emetic doses [implying that larger quantities are nauseating or emetic]; one-half to three-quarters of a grain, with a smaller amount of opium, every two, three or four hours, according to the case. Acetate of lead is also much employed," etc. Neither the auther nor his editor encourage its use. Dr. George B. Wood, after describing the treatment he thinks best, writes: (5th ed., ii., p. 633,) "Ipecacuanha has been thought to exercise a peculiarly favorable influence, and some practitioners have confided the cure chiefly to that remedy." He then briefly refers to "asserted" cures by large non-emetic doses. In his summary of the approved treatment of dysentery (p. 637,) he does not mention it. Flint, (*Practice*, 4th ed., 1873, p. 372,) who probably represents the most advanced general practice in this country, in sporadic dysentery "places reliance on purgatives and opium." Among the recognized but secondary methods, he says: "Ipecacuanha has been considered a valuable remedy in dysentery. Some have attributed to it a specific curative influence, and have advocated its employment in as large doses as can be borne," and then quotes Maclean. In his earlier editions he does not comment upon it, but in the latest, he reports: "I have resorted to this plan of treatment in a considerable number of cases in

Bellevue Hospital, but without a very satisfactory degree of success. In a small proportion of cases the disease was either arrested or favorably modified by it; but in the majority of cases it had no apparent influence on the disease." In epidemic dysentery he makes no reference whatever to it. Neither Meigs and Pepper (4th ed., 1870,) nor West (4th Am. fr. 5th Eng. ed., 1866,) refer to ipecacuanha in the dysentery of children, except in minute diaphoretic doses. Enough has been quoted to show that the plan here illustrated is probably followed by few practitionērs.†

Besides this, a careful study of the leading authorities in therapeutics who refer to its employment reveals that those who have not had personal experience with it look upon the drug as necessarily emetic, and at the treatment as essentially of that character. That this remarkable misapprehension is very common, the following quotations demonstrate:

Headland (*Action of Medicines*, 2d Am. ed., p. 283,) says "Ipecacuanha is in the first place a neurotic, in the second an eliminative." He then illustrates its neurotic character by its action as an emetic, and thinks it acts on the vagus and cannot act on the sympathetic for two reasons. 1. Because "other parts of this nerve would be influenced at the same time. But this does not appear to be the case." 2. "Such action could not be suddenly and violently evidenced, for the natural action of this nerve is slow, chronic and persistent." And he adds, "at all events the effects for which they are used could not be produced by an affection of this nerve." He considers it (p. 318,) to have a specific action on the bronchial mucous membrane, and to thus act as an eliminative. He speaks of it (p. 335,) as indirectly a diaphoretic when given in emetic doses, but makes no allusion to its possessing any influence upon the intestines. Pereira (3d Am. ed., 1854, ii., p. 624,) clearly limits its power in large doses to that of an emetic, and, referring to its former use in dysentery, says: "In severe forms of the disease no one, I suspect, would now think of relying on it as his principal remedy, but as an auxiliary

† Since its revival, ipecacuanha has been used in France in intestinal affections chiefly in the form of the Brazilian draught (*potion bresilienne*): viz. 10 grammes of the bruised root boiled in 200 grammes of water until it is reduced to 100 grammes, and then sweetened. (*Etude experimentale sur l'action therapeutique et physiologique de L'Ipecacuanha et de son Alcaloide*, par C. A. Polichronie, Docteur, etc., Paris, 1874, pp. 100; pp. 28-9.) This would not be non-emetic in its action.

its efficacy is not to be denied. Sir George Baker and Dr. Cullen consider it to be of most benefit when it acts as a purgative, but this can hardly be its *methodus methendi*." He regards (p. 628,) its efficacy to be due in part to its diaphoretic properties; "But its tendency to produce an anti-peristaltic movement of the intestines doubtless contributes to its anti-dysenteric properties." He then observes without comment, as if it were simply an isolated and peculiar fact that should be recorded, "Mr. Twining gave large doses (gr. vj.) with extract gentian without vomiting." Dr. G. B. Wood, (*Therapeutics*, 1856, ii., p. 435,) after describing it as an emetic, speaks of dysentery as a "complaint in which ipecacuanha has long enjoyed peculiar credit," and says "it has been given in it in two different methods: in one, in large doses with a view to its full emetic effect; in the other, in smaller doses, repeated so as to sustain a nauseating impression, or operate on the bowels." This is the first clear intimation we have of its intestinal influence. He then adds that Mr. Playfair's method "is to give, at the beginning of the attack, from thirty to sixty grains of the powder with as many drops of laudanum. If it vomit, the dose is to be repeated." Elsewhere (*Dispensatory*, 13th ed., p. 497,) he says, "Ipecacuanha is in large doses emetic, in smaller diaphoretic and expectorant. * * In quantities not quite sufficient to vomit, it produces nausea and frequently acts on the bowels." To this last property he does not appear to attach any special value. It is not unusual for therapeutists to speak of the relaxation of the bowels that often accompanies nausea as an effect of it. Is it not as rational to attribute the nausea and the catharsis to different manifestations of the same cause? Because they occur simultaneously, or nearly so, it is not necessary to suppose that the one creates the other. Later Dr. Wood remarks, "In dysentery it has been supposed to exert peculiar powers," but he does not intimate what those powers are nor how they are to be brought into action. And in treating of its uses, he gives no intimation that it may be employed in bowe affections, except as an emetic, otherwise than in doses of a quarter or a half-grain. Stille (*Therapeutics*, 3d. ed., 1868, ii., p. 390,) remarks that "in all doses it is apt to relax the bowels if they are in a healthy state," and "the laxative action of ipecacuanha, when given in small and repeated doses, cannot well be questioned.' He cites Piso, Murray and Richter as vouchers for its value in large doses in dysentery, but adds, "Pringle, however, thought that it succeeded best when it operated by stool as well as vomited. He indeed laid it aside on account of the distress produced by its action"—implying thereby that its primary, most powerful and normal action in dysentery is emetic. Dr. Stille thinks that when the testimony is narrowly examined it is evidently only of "advantage over other vegetable emetics or mild cathartics when the disease is epidemic or assumes bilious characters with bitterness of the tongue and a foul mouth." Further on in his article, however, he gives a full account of the Indian method; but, very strangely, under the head of Administration he only mentions its employment as an emetic, nauseant, expectorant and diaphoretic. Waring in his admirable treatise (*Practical Therapeutics*, 2d Am. fr. 3d. Lond. ed., 1871, p. 356,) gives a succinct and lucid account of its use in dysentery, crediting the paper chiefly to Professor Maclean, but offers no opinion as to its mode of action. He is careful to show that emesis is by no means a necessary consequence of taking twenty, thirty or more grains into the stomach; and he and Maclean are the only original writers that I have seen who do so state. They both appear to have had personal experience in its use. Dr. H. C. Wood, (*Therapeutics*, etc., 1874,) the latest systematic writer to whom I have had access, says, (p. 364,) "in large amounts it causes vomiting, accompanied * * by a decided increase of the secretions * * * of the salivary glands and of the mucous membrane of the bronchial tubes and of the stomach." "Whether given in large or small doses it is very apt to act upon the bowels, to increase and to modify their secretions." "In 'bilious dysentery' it will often produce large tarry discharges; * * the mechanical effect of the vomiting induced by it in these cases, however, must not be lost sight of; yet it does not seem to me at all sufficient to account for the result, especially as some observers state that the effects noted are produced even when little or no vomiting occurs." Treating of its use in this disease, he writes, (p. 368): "I think, however, its beneficial action is best seen in 'bilious dysentery' and in malignant dysentery, as is indicated by the fact that its use is most common in tropical climates." That the disease is more common in those climates and its treatment may be better understood there, is a plausible explanation of this fact. He continues: "In dysentery it is best to begin with a full emetic dose, or with ten grains, repeated every half hour until emesis is produced.

Two or three hours after vomiting, fifteen drops of laudanum should be exhibited followed in twenty minutes by five grains of ipecacuanha in *pill-form;* this should be repeated every two or three hours, the amount of the opium being lessened and that of the ipecacuanha increased, according to circumstances. The object is to have as much of the ipecacuanha retained as possible. Another plan is to give larger doses (twenty grains) repeated every two, four or six hours, mustard being applied to the epigastrium; and it is said that after two or three doses tolerance is established and the drug retained." It will be observed that the idea of primary emesis underlies this, the latest American teaching, as well as most of the others.

These quotations have been made at some length to show the drift of the authorities upon the treatment of dysentery and upon the action of ipecacuanha in that disease, and as the Apology for this paper.

But analysis of the cases herein reported demonstrates two facts with reasonable clearness. The first is the promptness with which the ordinary sporadic dysentery yields to large doses of ipecacuanha. The second is that ipecacuanha in twenty and thirty-grain doses is not necessarily followed by emesis.

Cases I., II., III., VII., XI., XV., XVIII., seven in all, were of well-marked dysentery, which yielded without delay to the influence of the drug. In neither of these cases was there a single bloody stool after the first dose was swallowed, and relief from the abdominal pain began promptly and was complete in from three to twelve hours.

Cases XII., XVI., XXIII., XXIV., four more, were of diarrhœa with a marked dysenteric tendency, which yielded with almost equal readiness, and in general terms might be classed with the preceding, making a dysenteric total of eleven.

In cases IV., V., IX., X., XIII., XIV., XVII., XIX., XX., XXI., XXII., eleven more, enteric pain, sometimes with and sometimes without diarrhœa, was relieved and natural stools were procured with varying degrees of promptness and of comfort.

Cases VI. and VIII. were of severe cholera morbus, one of which most certainly immediately responded to the drug, and the other either did likewise or there was a remarkable coincidence.

The facts concerning emesis are best shown in a table like the following. The tabulated doses were of twenty, twenty-five and thirty grains.

| Mode of Administration. | Retained perfectly. | Vomited within an hour. | Retained at least an hour, but vomited subsequently. | Total. |
|---|---|---|---|---|
| With opium and mustard.. | 16 | 2 | 8 | 26 |
| With opium only.......... | 20 | ............. | 9 | 29 |
| With mustard only........ | 3 | 1 | 3 | 7 |
| With no adjuvant.......... | 2 | 1 | 1 | 4 |
| Aggregate................. | 41 | 4 | 21 | 66 |

Besides these, four ten-grain doses were given to adults, a seven-grain dose to a child of thirty-four months, and two ten-grain doses to a child of seven years, being seven more, all but one of which were retained.

We thus find that seventy-three so-called emetic doses of ipecacuanha were administered, and that forty-seven of these provoked no vomiting whatever. Examination of the twenty-six cases where vomiting occurred shows at least two that, from the very trivial character of the ejection and from the provocations that induced it in each of them, may be transferred to the non-emetic list. These are: 1. Case VI., where two hours after the second dose had been taken, forty-five grains in all, a cup of tea was drank and a part of it was thrown up; 2. Case XII., where, three hours after taking twenty-five grains and having meanwhile sat up, "a little bitter fluid" was vomited. There were also two other instances, in case XVIII., where there was at least a foreign element of aid, but, as in these it is doubtful, they will be retained in the emetic list.

We find, then, that in forty-nine out of seventy-three consecutive instances, that were not selected and in many of which it was avowedly given as an experiment, practically no vomiting resulted from the medicine.

All the cases approaching dysentery yielded promptly to the treatment, and the only ones of this kind in which there was any vomiting were the XVth, after the character of the stools had changed and when the medicine was rashly given without both adjuvants; the XVIIIth, which see; and the boy's (XXIII) after the blood and straining ceased.

The only decided failure, in ordinary indisposition, to retain the medicine, proper care being taken in its administration, was in the case of the lady (XVII), who, after an hour's sleep, had a violent paroxysm of vomiting; and even here we may infer that the movement of the bowels and the general relief the next day was due to the drug.

Further, we find that of the twenty-four cases in which vomiting did happen, but four of them occurred within the first hour.

The other conditions observed in the series are not numerous enough to yield general deductions. Attention may be invited, however, to case XIII., where six grains was taken every six hours for three days, and afterward four three-grain doses for more than a fortnight, in none of which instances was there vomiting.

There are two methods of administration: On an empty stomach give from fifteen to twenty-five minims of the tincture of opium in a small quantity of water; fifteen or twenty minutes later apply a counter-irritant to the epigastrium, and at the same time give the powdered ipecacuanha in as little water as possible. With care a little more than two fluid-drachms of water will make thirty grains of ipecacuanha into a paste sufficiently liquid to be swallowed. Or, for those who take pills easily, that form may be employed. In such cases the opium, in the proportion of one grain to twenty, may be incorporated in the pill. Twenty-five grains of ipecacuanha can be put up into two boluses, or twenty grains of that drug and one of opium will make up in four pills. Laudanum might be used in the pill form of ipecacuanha, one advantage of which is that the local effect of the opiate is not dissipated before the other drug begins to be absorbed. Recumbent rest must be strictly maintained and no food nor drink be taken for at least four hours, and usually longer. The dose may be repeated in from two to six hours, or, should the first be rejected, the second may be given as soon as the stomach is settled. In India sixty and more grains at one dose have been retained. I incline to think that, where the stomach is empty, the medicine acts non-emetically in direct proportion to the severity of the attack. As a rule, patients should not be told the name of the medicine, in order to guard against its mental influence; and they should be warned to exercise self-control against incipient vomiting. I apprehend that some plan closely approaching that just indicated must be strictly followed, or the ordinary emetic overflow may occur, to the disgust of the patient and to the disappointment of the physician. In Dr. Woodson's experience, "vomiting followed in all the [nine] cases but one in from fifteen minutes to twelve hours." In these "fluids of all kinds were denied the patient for two or three hours before and after taking the medicine." I conceive that four hours is a better minimum of abstinence. But notwithstanding that disagreeable symptom they all promptly recovered. When we remember how mild the vomiting of ipecacuanha usually is, there are few patients who would not willingly endure it, if necessary, to escape the suffering and danger of an attack of dysentery. But I trust that it has been demonstrated that emesis is not a necessary result of this mode of treatment.

Notwithstanding the smallness of this series, I think that the results attained, confirmatory as they are of the earlier traditions and of the recent Asiatic experience, warrant, on purely empirical grounds, a greatly extended trial of this treatment in dysentery, and point to the restoration to the drug of its old title, "*radix anti-dysenterica.*"

In this connection attention is invited to the following modern reports of the use of this drug in affections of the bowels. The list does not profess completeness, but it may assist others who care to investigate the subject. Some Indian papers are: Docker, (*Lancet,** July, Aug., 1858, pp. 113, 169;) Cornish, (*Madras Med. Jour.,** Jan., 1861, p. 41; *Ranking's Abs.** xxxiii., p. 91;) Blacklock, (*Madras Med. Jour.,** Jan., 1861;) Donaldson, (*Edin. Jour.,** v., p. 583;) Ewart, (*Indian Ann. Med. Sci.,** 1863, p. 396; *Brit. For. Med.-Chir. Rev.,** xxxii., p. 58;) Cunningham, (*Edin. Jour.,** vii., p. 25). (None of the foregoing papers have I been able to examine.) Dr. Richard Whittingham, Surgeon Peruvian Navy, contributes a paper on its use in tropical dysentery (*Am. Jour. Med. Sci.,* 1860, xl., p. 379). In simple specific dysentery, either common or bilious, he gives from half a drachm to a drachm of the powder fasting or early in the morning, and says, "The medicine is *not* given as an *emetic* but to produce its specific action on the disease." He also gives an enema of ipecacuanha night and morning. The above is repeated for three days: or he gives ten grains with one grain of opium every six or eight hours, and uses vegetable astringents such as pomegranate root. Such cases are well in a week or ten days if seen early. In other varieties the treatment is modified accordingly. In the malignant form he relies on nux vomica and opium. The mortality in hospital is less than two per cent. of the cases seen before the colon is ulcerated. Dr. E. H. Janes (*Am. Med. Times*,* 1861, iii., pp. 28, 274,) gives an abstract of treatment of acute dysentery by large doses of this drug, with statistics of the result. Dr. A. A. Hœhling, U. S. Navy, (*Med. and Surg. Rep.*, 1868, xix., p. 327,) reports ipecacuanha as the chief reliance in the treatment of this affection in Chili, and cites a case as a type where a man ill five weeks was restored to health in seven days mainly by the use of this drug. Dr. John Stephen, of Reading, Penn., (*Med. and Surg. Rep.*, 1870, xxiii., p. 419,) reports three cases of severe acute dysentery cured immediately by ipecacuanha in doses of from thirty to eighty grains. These are well worth study, and are presented by that gentleman as examples "out of quite a number" that all yielded in the same manner to the same means. He considers it "as much of a specific in acute dysentery as is quinine in intermittent fever." Dr. A. P. Morrill (*Med. and Surg. Rep.*, 1870, xxiii., p. 503,) confirms the experience quoted and recalls the usual practice on this point fifty years ago in the Southern States. Dr. Dyce Duckworth (*St. Bartholomew's Hosp. Rep.*, 1871, vii., pp. 111–113,) quotes corroborative modern evidence from Mr. Hunter, Belize, Dr. Yandell, [Louisville?] Dr. Clark, London, and Mr. Eccles, Bombay, the last-named frequently using emetia as a substitute for the powder.†

In favor of the treatment of chronic dysentery with large doses of ipecacuanha, somewhat in the manner of that of the acute form, evidence is gradually accumulating. Dr. H. D. Bulkley (*Am. Medical Times*, 1862, iv., p. 64,) reports four cases of chronic dysentery and diarrhœa in which ten-grain doses were administered. Of these one patient vomited, but all were immediately relieved. Dr. Bulkley refers to Dr. McKidd's article, (*Edin. Med. Jour.*,* July, 1861,) where a diarrhœa of ten years' standing was promptly cured by the administration of ten to twenty grains every twelve hours. I have the impression, but cannot vouch for it, that certain forms of chronic diarrhœa are now habitually treated by large doses of ipecacuanha in at least one of the New

† The latest essay on this subject known to me is Polichronie's. That, arriving after the completion of this paper, has compelled certain additions to the text at the last moment. Attention is invited to it as probably the latest monograph on this subject. The author freely confirms all that has been said of the power of the drug over dysentery. See final note to this paper.

York hospitals. Dr. Bulkley's article contains this sentence: "It is said that the native doctors of Constantinople invariably give large doses of ipecacuanha in dysentery, and that their treatment of it is very successful." Dr. Willshire, (*Lancet*, 1862, ii., July, p. 62,) reports a grave case of chronic dysentery with severe exacerbations, which, after thirty days' unsuccessful treatment, immediately improved upon a prescription in which four grains of ipecacuanha three times a day held the chief place. Dr. W. E. Whitehead, U. S. Army, reported (*Pacific Med. and Surg. Journ.*,* iv., p. 11,) three cases treated successfully by large doses of ipecacuanha; and under date of August 24, 1874, he writes me, "Since that time I have treated many cases of chronic dysentery after the same plan and always with satisfaction to myself." To this I may add the memorandum of a case in Colorado, several years ago, of which I kept no notes, where, after exhausting the ordinary round of treatment, I supplied the patient, a civilian, with a number of twenty-grain powders, directing him to take one whenever an exacerbation came on. There was no attempt to use it systematically, but he was relieved for the time by one or two doses whenever he saw fit to employ them. It appears also (*Med. Record*, 1871, vi., p. 384, fr. *Med. Times and Gazette*,*) that, at the English Hospital, Metz, during the late Franco-German war, "the most obstinate and fatal disease the physicians had to deal with was dysentery of a very chronic character." After other methods "the ipecacuanha treatment was then tried, and with fair success; doses of five to fifteen grains of the powder were given three or four times a day; it rarely caused vomiting, except at the first dose, sometimes not even then." Dr. Duckworth (*St. Barth. Rep.*, 1871, vii., p. 116,) quotes Akenside as saying of the use of the drug, "*Neque interest utrum acuta sit dysenteria, an chronica: utrum sanguinem habeant dejectiones, an muco tantum constent.*"—(*De Dysenteria Commentarius*,* 1764, p. 39.) Duckworth (p. 113) reports a case of chronic dysentery cured by enemata of ipecacuanha, and further says (p. 117), "It is a fashion in India to employ an injection of the drug in dysentery.† * * The plan was tried in the hospital [St. Bartholomew's] in the case of a woman, * * who was sinking apparently from uncontrollable dysenteric diarrhœa, which ensued after an operation for strangulated femoral hernia." It was successful after the usual remedies had failed. "No nausea or vomiting occurred."

† See Dr. Whittingham's article already cited.

(This case will be referred to in a subsequent section of this paper.) He also quotes a successful case in a child reported by Dr. Hillier (*Med. Times and Gaz.*,* Jan., 1864,) but adds that when attempted "in those very obstinate cases of diarrhœa which occur when the lower bowel is the seat of tuberculosis and ulceration" it failed. But on the contrary we have the experience of M. Chouppe (*Practitioner*, No. lxiii., July, 1874, p. 58, fr. *Bul. Gen. Ther.*,* June 15, 1874,) who has used it by injection "with very satisfactory results in the diarrhœa of tuberculous patients and in the choleriform diarrhœa of young children.† In these cases "vomiting was never observed."‡ Of the seventeen cases of phthisical diarrhœa thirteen were cured and two improved.|| (*Sup. Med. News and Lib.*, Nov., 1874, p. 211.)

On the other hand there are two articles in the *Medical Times and Gazette* (1873, i., pp. 102, 194,) upon the failure of ipecacuanha in chronic dysentery. But no claim has ever been set up for its uniform success in that form of disease.§

Finally, attention is invited to a group of singular cases reported by Dr. James D. McGaughey (*Phil. Med. Times*, 1872, ii., p. 407,) as epidemic duodenitis. The treatment he found most useful was active purging, the free use of ipecac., cantharidal blisters over the tender points; and generally after free purgation, a thorough relaxation with ipecac., and a blister, the pain began to decrease. I cannot help suspecting that "the free use of ipecac." was an essential element of the cure.

(*Continued in next number of Journal.*)

---

† MM. Bourdon and Chouppe followed Trousseau in using the drug in infantile cholera, changing the administration from the mouth to the rectum. (Polichronie, *op. cit.*, p. 19.) M. Polichronie gives in detail M. Chouppe's five cases of infantile cholera and adds one by M. Huchard. There was one death in the six, which is attributed to errors of diet.

‡ Inflammation of the rectum, passing off when the treatment is suspended, frequently follows these enemata. M. d'Ornellas, using emetine, met with it in every case; M. Chouppe, using ipecac., found it in only five cases in thirty-four. (*LeProgres Medical*, No. 28, 11th July, 1874.)

|| Polichronie (*op. cit.*, pp. 29–31) gives details of one case of acute and three of chronic dysentery treated by enemata of ipecacuanha. One of the last was unsuccessful.

§ Maclean declares that many cases of the chronic form are not the result of unhealed ulcers, but are met with where no breach of surface is detected. The drug is indicated in an acute outbreak during a chronic attack. (Duckworth: *St. Bart. Rep.*, vii., 1871, p. 116.) Is it not possible that there is a flaw in the common teaching as to the essence of dysentery? On this subject more will be said later. Meanwhile hear Trousseau: "*l'ulcure est comme un bourg brule; ce n'est pas la guerre, c'est l'effet de la guerre.*"

